# Use of Formal and Informal Food Resources by Food Insecure Families in Lima, Peru: A Mixed-Methods Analysis

**DOI:** 10.1007/s10900-021-00989-y

**Published:** 2021-04-27

**Authors:** J. D. Brewer, M. P. Santos, M. A. Lopez, V. A. Paz-Soldan, M. P. Chaparro

**Affiliations:** 1 School of Public Health and Tropical Medicine, Department of Global Community Health and Behavioral Sciences, Tulane University, 1440 Canal Street, New Orleans, Louisiana 70112 USA; 2 School of Public Health and Tropical Medicine, Department of Biostatistics and Data Science, Tulane University, 1440 Canal Street, New Orleans, Louisiana 70112 USA; 3 Asociación Benéfica PRISMA, Avenida Santo Toribio 115, 5th floor, San Isidro, 15073 Lima, Peru

**Keywords:** Food insecurity, Food assistance programs, Food resources, Coping strategies, Peru

## Abstract

The goal of this study was to measure food insecurity among families with children in a low-income district of Lima, Peru and to identify the formal and informal food resources available to them that may affect their food security status. In June-July 2019, we collected data from 329 randomly selected households in Villa El Salvador (Lima, Peru). Following a mixed methods approach, we found that the percentage of households using food assistance programs (FAPs) increased with increasing levels of food insecurity, but two FAPs were heavily used by households regardless of food (in)security. The main reasons for using FAPs included financial need, already being signed up in the program, and believing that the food was of nutritional value; the main reasons for non-use were finding the program unnecessary, dislike or poor perceived quality of the food, and not being able to sign up for the program. Similarly, informal food resources, such as buying food on credit or receiving food from someone outside the household, were incrementally used with increased levels of food insecurity. Our study clarifies the relationship between level of household food insecurity and FAP use – FAPs more commonly used by food insecure households were used because of financial need, whereas the FAPs most commonly used by food secure households were those with automatic enrollment. At a programmatic level, our research highlights the need for making nutritious and preferred foods available in FAPs and standardizing the application of enrollment criteria.

## Introduction

Food security “exists when all people, at all times, have physical and economic access to sufficient, safe, and nutritious food to meet their dietary needs and food preferences” [1]. Food insecurity, thus, refers to the inability to acquire adequate nutritious foods due to economic constraints and can be measured on a continuum as mild, moderate, or severe. In 2019, 2000 million (over 25%) of people worldwide experienced moderate or severe food insecurity [2]. In Latin America, 191 million people experienced moderate or severe food insecurity in 2019, representing 31.7% of the region’s population, up from 22.6% in 2014 [2]. Food insecurity has been linked to a number of negative nutritional outcomes, such as wasting and underweight, stunting, and overweight/obesity, depending on the specific context and the age group under study [3]. The type of food assistance that families have access to, both formal (i.e. food assistance programs; FAPs) and informal (i.e. buying food on credit, exchanging goods and services for food), can contribute to their risk of food insecurity and/or be used to mitigate the effects of food insecurity.

Both FAP use and use of informal food resources have a bi-directional relationship with food insecurity – these resources are more likely to be used by those who are food insecure, but their use can also lead to decreased food insecurity. For example, in countries such as Uruguay, Mexico, and the United States, studies have found use of FAPs is more common among food insecure households and individuals [4–8]. However, research in Mexico and the United States has shown that participation in FAPs can lead to decreased household food insecurity [9, 10]. In terms of informal food resources, previous research has shown that greater social capital and being a member of an organization were associated with decreased risk for food insecurity, since families with these characteristics frequently participated in meal sharing [11–13]. In contrast, studies in rural parts of Ethiopia, Nigeria, and the United States found that food insecure households are more likely to borrow money than food secure households [13–15].

In Peru, food insecurity is not routinely measured in national health surveys. However, a recent summary from the *Young Lives* study, an ongoing cohort study of families with children in 20 urban and rural districts in Peru, reported that food security increased from 27% in 2009 to 44% in 2013, with an associated decrease in mild and moderate food insecurity [16]. However, severe food insecurity increased from 7.5% to 11% in the same time period, and this increase was driven by urban poor families [16]. The Peruvian government funds several FAPs to address food insecurity and other nutritional concerns. These operate primarily in low-income neighborhoods and are partly managed by community members.

Noteworthy FAPs include: *Comedor Popular*, a community kitchen program which provides low-cost meals to people living in poverty; *Vaso de Leche*, or “Glass of Milk”, which distributes rations of 250 mL of fortified dairy- or grain-based milk to families with young children and other vulnerable individuals; *Cuna Más*, an early childhood development program that provides childcare and two meals per day to young children; and *Qali Warma,* the school feeding program, which provides a breakfast of a drink (dairy-, fruit-, or grain-based) and a baked product or a lunch of a non-perishable food item (grain, tuber, or protein-based preserve) to children in public primary schools [17–20]. Even though *Comedor Popular* and *Vaso de Leche* target families living in poverty in low-income neighborhoods, there is no hard criteria to enroll beneficiaries. Participants are selected at the discretion of local mother’s committees who administer the programs, without the need for earnings’ documentation. Additionally, at *Comedor Popular*, if there are leftover meals after enrolled participants have eaten, non-participants can purchase a reduced-cost meal. On the other hand, *Qali Warma* automatically enrolls all children in schools where the program is available, whether or not the individual child has a need for the program, though the program is only available in public schools in low-income neighborhoods. For a detailed breakdown of program eligibility criteria, see Table 1. At the community level, research has recorded a number of informal coping strategies for food access in Peru, such as buying food on credit [21] or borrowing money for food [22], bartering services for food [21], and hosting *polladas* (community-organized fried chicken sales for the purpose of fundraising) [23].

**Table 1:** Compared eligibility for Food Assistance Program (FAP) use according to program’s target population, questions included in the questionnaire, and study data analysis plan

Food assistance program	Program target population	Question on FAP participation asked if:	Coded as eligible to participate if:
Comedor popular	People living in poverty/extreme poverty. Beneficiaries chosen at discretion of mothers’ committees in neighborhood; non-participants can receive leftover meals at reduced cost, if available	Previously answered that there was a *Comedor Popular* in their neighborhood	Answered positively that there was a *Comedor Popular* in their neighborhood
Vaso de Leche	1st priority: children under the age of 6, pregnant and lactating women. 2nd priority: children aged 7–13, elderly, tuberculosis patients; poor/extreme poor. Beneficiaries chosen at discretion of mothers’ committees in neighborhood	For all children in household under the age of 18	Households with at least one child under the age of 14^a^
Cuna Más	Children under the age of 3 who live in poverty	For all children in household under the age of 3	Households with at least one child under the age of 3
Qali Warma	Children enrolled in public kindergartens and primary schools	For all children aged 6–18 years old for whom the respondent previously answered positively regarding the child’s school participating in *Qali Warma*	Households with at least one child aged 6-13^b^

^a^While we inquired participation for all children (< 18 years old), only the 1st and 2nd priority groups of minors (< 14 years of age) was considered eligible, as only two children over 13 years received *Vaso de Leche*

^b^Only school-aged children were considered eligible, as *Qali Warma* is served only in primary school (6–13 years old) and only 3 non-school aged children reported participating in *Qali Warma*

Research connecting food security with the use of these formal and informal food resources in Peru is lacking. To our knowledge, no studies have evaluated how FAP use is associated with food insecurity in Peru. Similarly, studies on the connection between informal food resource use and food insecurity are limited – two studies in Peru have found that social connectedness or capital were associated with food security [24, 25] and only one in the Amazon Region specified how certain coping strategies, such as gifting and receiving food, improved food security [21]. Given that severe food insecurity in Peru is increasing particularly among the urban poor [16] and that people living in urban poverty have unique experiences and challenges related to food insecurity [26], the goal of this study was to identify the formal and informal food resources available to, and used by, food insecure families living in an urban poor district of Lima, Peru. This research is increasingly pertinent as economic access to food and FAP accessibility have been limited by the COVID-19 pandemic in Peru [27].

## Methods

### Study Setting

Our study took place in Villa El Salvador (VES), a low-income district in Lima, the capital of Peru. VES is the fifth largest district in Metropolitan Lima, with approximately 463,014 residents in 2015 [28], 20–25% of whom lived in poverty in 2017 [28]. VES is located in the southern cone of Metropolitan Lima, which accounts for a large proportion of households of low socioeconomic status compared to the city center, a distinction linked to a historical concentration of migration in the city’s peripheral districts [29]. VES started as an informal settlement of squatter communities who did not have formal ownership of their land; however, residents organized to obtain legal land titles and public services over time [29, 30]. This social movement also manifested in women’s and mothers’ committees, which worked to create precursors to government-funded FAPs, such as *Vaso de Leche* and *Comedor Popular* [31].

### Study Design

In order to identify the food resources available for, and utilized by, food insecure families, we collected data via a household questionnaire. We estimated that a sample of 450 participant households was necessary to detect a prevalence of food insecurity of 56% [16] with a power of 80% and margin of error of 5%, assuming an 80% complete response rate. Given that poverty is the main risk factor for food insecurity, we wanted to evaluate how food insecurity and food resource availability and utilization differed across households of different income strata. Therefore, we randomly sampled 150 neighborhood blocks from each of the three income strata present in VES: low income (average monthly income less than US $164), lower-middle income (average monthly income US $164–256), and middle income (average monthly income US $256–379) [32]. We recruited one household per neighborhood block via systematic sampling until we found a household that fit the eligibility criteria and consented to participate in the survey. Eligibility criteria included that the respondent had to be in charge of food purchase in the home, be at least 18 years of age, and have at least one child (< 18 years old) living in the household.

### Data Collection

All data were collected between June-July 2019. The research team included three experienced local data collectors plus three US-based research assistants with training in public health research. We attempted recruitment with 2267 households; over half of these households did not answer the door or were ineligible to participate. Of the eligible 714 with which we made contact, 329 households participated in and completed the questionnaire, for a cooperation rate of 46.1%; 104 of the households that completed the survey were from lower-income neighborhood blocks, 121 from lower-middle income blocks, and 104 from middle income blocks.

### Study Instrument

The household questionnaire included demographic and socioeconomic questions, the Household Food Insecurity Access Scale (HFIAS), and questions on FAP participation and informal methods of food access. The questionnaire was first piloted for validation with 19 conveniently-sampled households in the district of San Juan de Lurigancho, which has similar socioeconomic characteristics as VES.

To measure the presence and severity of food insecurity, we utilized HFIAS, an internationally validated 18-question scale [33] which has been translated into Spanish and validated in previous studies in Peru [34]. The section on FAP participation and informal methods of food access was designed by our study team based on similar studies measuring food resources in Peru [21, 25, 35]. Respondents were asked if they participated in any of the following FAPs: *Comedor Popular, Vaso de Leche, Cuna Más*, and *Qali Warma*. The respondent was asked if they had used the programs only if they met the eligibility criteria outlined in Table 1. Respondents were then asked open-ended questions about the main reasons why they reported using the program (if reported use was within 6 months prior to the survey) or not using the program. Even though questions about FAP use and reasons for use/non-use were asked for each eligible child in the household, we report use and reasons for use/non-use at the household level.

For informal food resources, participants were asked if in the 7 days prior they did any of the following: purchased food on credit, borrowed money to purchase food, received food for free from someone who is not a member of their household (excluding food obtained through FAPs), or prepared food with someone who is a not a member of their household. Respondents were asked about these informal food resources over the previous 7 days because of the assumption that these methods may be more difficult for respondents to recall over a longer period of time.

### Quantitative Data Analysis

We ran descriptive statistics (i.e. frequencies) to summarize the use of formal and informal food resources among eligible respondents according to severity (mild, moderate, severe) of household food insecurity (Table 2). Use of formal food resources (i.e. FAPs) was estimated both for the previous 4 weeks to match the timeframe of the food insecurity questions, and for the previous 6 months to match the timeframe of the qualitative responses. Use of informal food resources was estimated for the previous 7 days as that was the only timeframe measured. Additionally, we ran chi-square tests to identify significant differences in the use of FAPs and informal food resources by severity of food insecurity, with a p-value of < 0.05 considered statistically significant.

**Table 2:** Use of formal and informal food resources among eligible respondents by severity of household food (in)security, Villa El Salvador (Lima, Peru)

	Total eligible, N	Food secure (%)	Food insecure		
			Mild (%)	Moderate (%)	Severe (%)
Interviewed households	329	22.8	14.9	24.0	38.3
Formal food resource use in past 6 months^a^					
Comedor Popular	137	40.0	58.3	58.3	62.5
Vaso de Leche*	298	21.2	23.4	36.0	42.7
Cuna Más	141	0	5.0	5.0	6.1
Qali Warma	237	42.0	40.5	36.0	52.0
Formal food resource use in past 4 weeks^a^					
Comedor Popular	137	28.0	33.3	41.7	37.5
Vaso de Leche*	298	19.7	21.3	34.7	37.3
Cuna Más	141	0	5.0	5.0	6.1
Qali Warma	237	42.0	40.5	36.0	50.0
Informal food resource use in past 7 days					
Purchased food on credit***	329	9.3	14.3	38.0	53.2
Borrowed money to purchase food***	329	2.7	4.1	16.5	31.0
Received food from someone outside of household**	329	14.7	18.4	38.0	29.4
Prepared food with someone outside of household	329	20.0	16.3	21.5	24.6

^a^Percentages are out of households eligible for the FAP, by food (in)security level

Statistical significance based on Likelihood Chi-Square is denoted by:

*p < 0.05

**p < 0.01

***p < 0.001

### Qualitative Data Analysis

Each step of the qualitative data analysis process was conducted separately for each open-ended question, creating a unique codebook for use and non-use of each FAP. The primary coder [JDB] developed the first version of the codebooks through line-by-line coding of all data points, following a grounded theory approach [36]. Codes were validated with expert-checking, presenting the codebooks and example quotes to the study’s local data collectors, who used their familiarity of the study location and FAPs to confirm the code relevance and suggest changes. The primary coder then used this feedback to edit the codebooks and re-code all data. The primary coder combined or divided codes for additional clarity, producing the final versions of the codebooks. At this point, the primary and secondary coder [JDB, MAL] coded all data with the finalized codebooks using qualitative analysis software NVivo 12, blind double-coding every fifth data point. The coders attained kappa scores ranging from 0.74 to 1.00 for each question codebook, demonstrating high inter-rater reliability. Qualitative results for *Cuna Más* are not included as only 6 respondents participated in this program.

## Results

### Quantitative Results

In general, the use of formal food resources increased as severity of food insecurity increased, though the only significant difference was observed for *Vaso de Leche* (Table 2). The most commonly used FAP for food insecure households in the 6 months prior to the survey was *Comedor Popular*. When measured in the previous 4 weeks, this shifted to *Qali Warma* for all levels of food insecurity except moderately food insecure. The most commonly used FAP for food secure households was *Qali Warma*, at both 6 months and 4 weeks. Interestingly, use of FAPs was highly prevalent among food secure households, who heavily relied on both *Qali Warma* and *Comedor Popular.* The least used FAP across all levels of severity of food (in)security was *Cuna Más.*

Use of informal food resources also increased with increased severity of food insecurity; this observed increase was statistically significant for all categories except for “prepared food with someone outside the household” (Table 2). The most commonly used informal food resource varied at each level of food (in)security: food secure households tended to prepare food with someone outside the household, mildly food insecure households reported receiving food from someone outside the household, moderately food insecure households reported both receiving food from someone outside the household and purchasing food on credit, and severely food insecure households tended to purchase food on credit.

### Qualitative Results

The most common reasons for using or not using FAPs varied by FAP. Below, we report the most frequently occurring themes for use and non-use for each FAP.

#### Comedor Popular

Of the reasons that respondents gave for utilizing *Comedor Popular*, the most frequently occurring themes were **financial need** and **time**. For example, when asked the main reason they attended the *Comedor Popular*, one respondent shared that they went, “when money gets tight for cooking” and another said because, “I save on gas and oil.” Other responses related to being unable to cook their own food, “due to a lack of time to cook”, feeling rushed, not being in the house to cook, or simply not wanting to cook.

Alternatively, the most frequently occurring themes for why respondents did not use *Comedor Popular* included feeling that it was **unnecessary**, i.e., they perceived they had no need to participate in the program, and due to the quality of the **food** itself. In regards to finding the program unnecessary, some respondents simply explained, “There is no need,” while others elaborated, “I work and have enough to cook,” and, “It is for people who need it more.” With respect to the quality of the food, a few respondents stated, “It is not the same what I cook” and “We do not like the food.” Others claimed, “The food is not nutritious.”

#### Vaso de Leche

The most frequently occurring themes for using *Vaso de Leche* were that respondents were **already signed up**, **financial reasons,** and because they believe the food provided **nutritional value**. A vast majority of respondents stated the main reason they participated in the program was because they were already signed up and received the food, or because they felt it was their right to receive the food. Many respondents explained they received *Vaso de Leche*, “for [the child’s] breakfast” or “because of [the child’s] age and because she is signed up.” Others specified that they received *Vaso de Leche*, “because the government gives it to [the child].” Financial reasons ranged from feeling a need for the ration (e.g. “It is necessary for me, and sometimes there is no money,”) to finding it advantageous compared to buying food (e.g. “It is in our best interest instead of buying milk”). In terms of the nutritional quality of the food, many respondents emphasized the presence of oatmeal in the food ration, with a few specifying, “It is good to eat oatmeal.” Others spoke generally of the nutritional value of the food, “It reinforces [the child’s] diet.”

Alternatively, the most frequent themes for not using Vaso de Leche were related to not being registered for the program. First, respondents stated that they were **not allowed to sign up** or were deemed ineligible for various reasons: “They did not let me sign up,” or “Due to [the child’s] age, they no longer give it to him,” or “They did not want to give it to me because I have a fully constructed house [measure of socioeconomic status].” The second most common reason for not using Vaso de Leche was simply **not being signed up at the present moment**, but without evidence that they were not allowed to sign up or had tried to and were unable. Many respondents simply responded, “We are not signed up,” while some added, “They do not come to sign us up.” Following reasons related to registration, respondents also gave reasons related to the **food** (e.g. “They always prepare the same thing,” and, “I do not think it is healthy food”) and feeling that the program was **unnecessary** for them (e.g. “Here at home we have food”).

#### Qali Warma

The overwhelming amount of responses for why respondents’ children consumed foods from *Qali Warma* were because the children were **given the food** because the child is in a school with the program: “They give it to [the child] in school,” and, “They give it to all children,” while some specified, “They are in school and the government has an obligation to give it to them.” The following most frequent themes for consuming *Qali Warma* had to do with an aspect of the food, either **liking the food** (e.g. “[The child] like the cookies and the milk too”) or expressing that it has **nutritional value** (e.g. “They give it to [the child] for its vitamins,” and, “For nourishment”).

Of the 107 respondents who said their children attended a school with *Qali Warma*, only nine said their child did not eat the food. Seven out of the nine responses for the main reason the child did not eat the food were related to an aspect of the **food**, either a dislike for the food (“[The child] does not like it”) or doubting the quality of the food, (“He does not eat it because he gets food that is in poor condition”).

## Conclusions

Our study aimed to identify the formal and informal food resources available to, and used by, food insecure families living in an urban poor district of Lima, Peru. Our findings add to a body of evidence from other countries that food insecure households have high participation rates in FAPs compared to food secure households [4, 6–8]. However, we also found heavy use of FAPs among the food secure. This could be because all households under study were from a low-income neighborhood, thus even those households that were food secure were not wealthy and had easy accessibility to FAPs.

Similarly, our results showed increasing proportions of households using informal food resources at each increasing level of food insecurity. This differs from previous findings in Peru that showed informal food resource use or similar intangibles were associated with decreased food insecurity [21, 24, 25]. This may be because two of these studies focused on social networks, rather than specific coping mechanisms, [24, 25] and the third, which assessed gifting and receiving food, was conducted in the Amazon Region where practices around food sharing may be different [21]. Our findings are consistent with studies in other parts of the world that show that use of informal food resources, such as borrowing money, increases with increasing food insecurity [13–15]. Given the flexible nature of some FAPs in Peru, the use of informal food resources may be more telling of a household’s food insecurity, specifically purchasing food on credit and borrowing money to buy food, which steadily increased with increasing levels of food insecurity in our study.

Our qualitative findings of reasons for FAP use and non-use were consistent with the literature. Other qualitative and mixed-methods studies in Argentina, the US, Canada, and the UK support our findings that financial need is a principal reason for FAP use [13, 37–39]. Research in Argentina, Brazil, and the US supports our findings of ineligibility, lack of access, and other system hassles as primary barriers to FAP participation [13, 37, 40]. Additionally, research in Canada and of Latinos living in the US found that not having access to preferred foods limited participants’ abilities to use food banks as many participants desired foods of better nutritional quality [38, 41]. This is comparable to our findings that concerns about food preference and nutritional quality were primary reasons for not using FAPs.

By using mixed methods, our study explored relationships between food (in)security and food resource use that may not be captured in research relying on only quantitative or qualitative data. For example, financial need was a main reason for using both the *Comedor Popular* and *Vaso de Leche*, and we saw that use of these programs tended to increase with increasing severity of food insecurity. This may suggest that these two programs are reaching their target population of those with financial need. Alternatively, most people reported their children consumed food from *Qali Warma* because it was given to them in school, not necessarily because of financial need. This could explain why food secure households most frequently used *Qali Warma*. The different qualitative and quantitative results for these programs can be explained by their structures—*Comedor Popular* and *Vaso de Leche* require individuals to sign up, whereas *Qali Warma* is given out to all children in public schools where it is present, regardless of individuals’ needs.

This study shows some possible areas of intervention to make FAPs more effective in addressing food insecurity in urban areas of Peru. Issues related to food were prominent reasons for non-use across FAPs. It is important that FAPs provide food that is both nutritious and fitting of beneficiaries’ tastes and preferences. Research has found that some foods provided by FAPs in Peru are nutritionally insufficient [20, 42–45] and simultaneously high in carbohydrates, sugars, and saturated fat, [42, 43] but research on beneficiaries’ perceptions of these foods is more limited [45, 46]. Additionally, there were particular concerns with *Vaso de Leche* about respondents being considered ineligible or not allowed to sign up for other reasons. Though many respondents did not participate because they did not meet standard program eligibility criteria, there seemed to be inconsistent application of this criteria (for example, respondents cited different age cut-offs) or application of subjective criteria (e.g. owning a business or having a fully constructed house as evidence of socioeconomic status that precludes participation). Furthermore, many respondents were not given any reason for why they could not participate. This confusion around eligibility is likely due to the fact that, though the government sets forth criteria for first and second priority target populations, the final decision on enrollment is at the discretion of local mothers’ committees, so application of criteria is subjective. Previous studies have shown that actual users of various FAPs in Peru do not always meet government eligibility criteria [20, 44, 45, 47–49]. The flexibility around eligibility criteria in Peru is an important distinction from the more standardized practices in countries where the overwhelming majority of research on FAPs takes place, and thus needs to be considered in both scientific studies and policy proposals.

This research has a number of strengths. To our knowledge, it is the first study in Peru to assess FAP use by level of food (in)security, and one of the first to investigate this and the use of informal food resources among the urban poor in Peru. It used a stratified random sample; thus the data can be considered representative of the low-income district under study and possibly other similar districts within Lima. Finally, qualitative themes about FAP use/non-use were validated via expert-checking with local data collectors.

However, the study also needs to be seen in light of certain limitations. First, different timeframes were used to measure FAP and informal food resource use (past 6 months or past 4 weeks, and past 7 days respectively), which may complicate comparisons between the use of these resources. Different timeframes were assigned to FAP and informal resource use to replicate similar questions in previous studies in Peru [21, 35] and because of the assumption that it may be harder to recall informal food resource use beyond 7 days, whereas FAP use is usually continuous. A second limitation was that all qualitative data came from a small series of short-answer questions, nested within a 30-min household questionnaire where answers were typed by data collectors into a tablet. Compared to voice recordings, this form of data collection may have resulted in the loss of some level of detail or been susceptible to observer bias. Additionally, qualitative data from a survey format is naturally more limited than other qualitative methods, like in-depth interviews, which could more naturally capture narratives of how participants began using FAPs. Third, slightly different wording between open-ended questions for each FAP may have led to responses that are hard to compare across FAPs. For example, we asked, “What is the main reason you go to the *Comedor Popular*?” compared to, “What is the main reason the child eats *Vaso de Leche*?” The latter question focuses on consuming the *Vaso de Leche* ration and not the reason for household participation in order to allow for separate responses for each child in the household, not only eligible children. This was to capture household sharing of *Vaso de Leche* food rations with non-eligible family members, which we found in our study and has been cited in the literature [46–48]. Finally, the differences between FAP eligibility criteria for our questionnaire and data analysis, as well as from program standards (Table 1), may have created some discrepancies in analysis. The purpose for these distinctions was to capture as large a breadth of FAP users from the questionnaire, but then minimize the number of eligible respondents for analysis so as to not inflate non-use. Given the flexible and inconsistent application of program criteria, [47, 48] we felt that only surveying respondents who fit program criteria would result in excluding a considerable number of families who used the FAP despite not being eligible.

Future studies would benefit from creating a purposive sample of households that are eligible for or participate in *Cuna Más*, as the number of *Cuna Más* participants was negligible in our study. It would be valuable to capture the state of food (in)security among *Cuna Más* beneficiaries as well as collect qualitative data on why eligible families do or do not participate in the program. This would allow researchers and policymakers to understand if *Cuna Más* is reaching its target population and possible barriers or enablers to program use. Additionally, given the predominance of “food quality” as a theme for both FAP participation and non-participation, this study could be complemented by further research on beneficiaries’ perceptions of the quality of, and their preferences for, the food offered at FAPs in Peru. The food security construct includes not only quantity of food available, but also access to nutritious foods that meet food preferences [1]. Therefore, if FAPs aim to address issues of food insecurity, it is important that researchers and policymakers alike pay attention to what kind of food is available in these programs and what beneficiaries think of them.

Further, the ongoing COVID-19 pandemic and associated economic crisis has highlighted the urgency to identify alternative ways for governments to provide food to those in need. For large parts of 2020, FAPs in Peru were not able to operate in normal capacity due to shutdowns and social distancing measures. Reconsidering the distribution patterns of traditional food assistance is thus warranted. Moreover, in times of crises such as the current one, informal food resources may become significantly more important than FAPs at helping people deal with food access issues. Future studies should further investigate these types of communal assistance as important coping mechanisms for food insecure families living in urban poverty in times of crisis.

## Data Availability

The original data collected in this study can be made available at the request of the journal and/or reader.

## Code Availability

The most frequently occurring qualitative codes, example quotes, and range of kappa scores for the codebooks can be found in the manuscript. Additional information, such as code definitions, code frequency, and specific kappa scores, can be made available at the request of the journal and/or reader.
